# Attaching DNA to Gold Nanoparticles With a Protein Corona

**DOI:** 10.3389/fchem.2020.00121

**Published:** 2020-02-25

**Authors:** Rong Wu, Huaping Peng, Jun-Jie Zhu, Li-Ping Jiang, Juewen Liu

**Affiliations:** ^1^State Key Laboratory of Analytical Chemistry for Life Science, School of Chemistry and Chemical Engineering, Nanjing University, Nanjing, China; ^2^Department of Chemistry, Waterloo Institute for Nanotechnology, University of Waterloo, Waterloo, ON, Canada; ^3^Higher Educational Key Laboratory for Nano Biomedical Technology of Fujian Province, Department of Pharmaceutical Analysis, Faculty of Pharmacy, Fujian Medical University, Fuzhou, China

**Keywords:** biosensors, aptamers, nanomaterials, biointerface, bioconjugation

## Abstract

DNA-functionalized gold nanoparticles (AuNPs) have been widely used in directed assembly of materials, biosensors, and drug delivery. This conjugate may encounter proteins in these applications and proteins may affect not only DNA adsorption but also the function of the attached DNA. Bovine serum albumin (BSA) with many cysteine residues can strongly adsorb on AuNPs and this conjugate showed high colloidal stability against salt, acid and base. Similar protection effects were also observed with a few other common proteins including catalase, hemoglobin, glucose oxidase, and horseradish peroxidase. DNA oligonucleotides without a thiol label can hardly displace adsorbed BSA, and BSA cannot displace pre-adsorbed DNA either, indicating a strongly kinetically controlled system. Thiolated DNA can be attached at a low density on the AuNPs with a BSA corona. The BSA corona did not facilitate the hybridization of the conjugated DNA, while a smaller peptide, glutathione allowed faster hybridization. Overall, proteins increase the colloidal stability of AuNPs, and they do not perturb the gold-thiol bond in the DNA conjugate, although a large protein corona may inhibit the hybridization function of DNA.

## Introduction

DNA-functionalized gold nanoparticles (AuNPs) (Brewer et al., 2005; Giljohann et al., 2007; Patel et al., 2011; Cutler et al., 2012; Pei et al., 2012; Xiang et al., 2013; Wang et al., 2017; Liu and Liu, 2019) have been extensively studied in terms of preparing the conjugate (Liu and Liu, 2017b), sensing (Wu et al., 2018), making materials (Tan et al., 2014; Jones et al., 2015), and cell-related applications (Giljohann et al., 2010). All the four DNA bases can strongly adsorb on gold via coordination interactions (Liu et al., 2018b), and thus even non-thiolated DNA can be attached to AuNPs (Kimura-Suda et al., 2003; Pei et al., 2012; Zhang et al., 2012a,b).

The adsorption of DNA is affected by many factors such as salt or ionic strength (Storhoff et al., 1998; Cutler et al., 2012). Since both DNA and AuNPs are negatively charged, salt is needed to screen the long-ranged charge repulsion to enable DNA adsorption. In addition, DNA adsorption is also promoted at low pH (Zhang et al., 2012b) and by freezing (Liu and Liu, 2017a). We also noticed the effect of various anions such as bromide (Liu et al., 2018b), and arsenate (Zong et al., 2019), and small molecules (Wu et al., 2019), which compete with DNA for the adsorption sites on gold.

Proteins represent another important type of molecules. DNA/AuNP conjugates used in biological applications such as intracellular delivery (Casals et al., 2010; Giljohann et al., 2010; Raeesi et al., 2016) would encounter blood and intracellular proteins. Proteins are different from small molecules since proteins can attach to AuNPs via polyvalent interactions (Albanese and Warren, 2011; Goy-Lopez et al., 2012; Dominguez-Medina et al., 2013; Chaudhary et al., 2014). The interaction forces can be classified as electrostatic attraction (Brewer et al., 2005; Casals et al., 2010; Cañaveras et al., 2012; Goy-Lopez et al., 2012) and stronger chemisorption via cysteine and lysine residues (Sen et al., 2011; Tsai et al., 2011). In addition, adsorbed proteins may experience time-dependent conformational changes on AuNPs (Tsai et al., 2011) leading to irreversible protein denaturation (Casals et al., 2010).

We were curious about the relative adsorption strength of DNA and protein. Considering the large size of both proteins and DNA and their strong adsorption affinities, kinetic effects may dominate. Bovine serum albumin (BSA) is a commonly used model protein and we employed it in this study. BSA was reported to enhance the stability of AuNPs even in cell culture media (Alkilany et al., 2009; Albanese and Warren, 2011; Dominguez-Medina et al., 2013; Tebbe et al., 2015). A few other proteins and a small peptide, glutathione (GSH) were also included for comparison.

## Experimental

### Materials

All of the DNA oligonucleotides were from Integrated DNA Technologies (IDT, Coralville, IA, USA; see Table 1 for sequences). Sodium citrate, potassium cyanide (KCN), and 4-(2-hydroxyethyl) piperazine-1-ethanesulfonic acid (HEPES) were from Mandel Scientific (Guelph, ON, Canada). BSA, FITC-labeled BSA (FITC-BSA), catalase (CAT), hemoglobin (Hb), glucose oxidase (GOx), horseradish peroxidase (HRP), hydroxy-1-hexanethiol (MCH), and L-glutathione reduced (GSH) were from Sigma-Aldrich. The 13 and 24 nm AuNPs were prepared using the citrate reducing method (Liu and Lu, 2006), and these were called citrate-AuNPs. Milli-Q water was used to prepare all of buffers and solutions.

**Table 1:** DNA sequences and modifications.

**ID**	**DNA names**	**Sequences 5′-3′**
1	FAM-9A5-SH	SH-AAAAAAAAACCCAGGTTCTCT-FAM
2	9A5-SH	SH-AAAAAAAAACCCAGGTTCTCT
3	FAM-cDNA	GGGTCCAAGAGA-FAM
4	FAM-rDNA	TCACAGATGGGT-FAM
5	FAM-A15	FAM-AAAAAAAAAAAAAAA
6	FAM-T15	FAM-TTTTTTTTTTTTTTT
7	A15	AAAAAAAAAAAAAAA
8	T15	TTTTTTTTTTTTTTT

### Instruments

UV-vis absorption spectra were collected on a spectrometer (Agilent 8453A). The ζ-potential and size distribution data were recorded using dynamic light scattering on a Zetasizer Nano 90 (Malvern). The fluorescent intensity was recorded on a microplate reader (SpectraMax M3) with excitation at 485 nm and emission at 520 nm. Adsorption kinetics were collected by a Cary Eclipse fluorometer in a quartz fluorescence cuvette.

### Preparation of AuNP/Protein Conjugates

To obtain AuNP/protein conjugates, 10 μL of HEPES buffer (100 mM, pH 7.4) was added into 50 μL of the as-synthesized 13 nm AuNPs (10 nM). Then, 10 μL protein (BSA, CAT, Hb, GOx, or HRP) with different concentrations was added and the final volume was adjusted to 100 μL followed by 30 min incubation. The AuNP/protein conjugates were washed by 10 mM HEPES buffer (pH 7.4) after centrifugation. For the larger 24 nm AuNPs, the concentration of BSA was 30 μM.

### Comparison of Adsorption Between DNA and BSA

The AuNP/FAM-A_15_ and AuNP/FAM-T_15_ conjugates were obtained by mixing 3 μM DNA and 10 nM 13 nm AuNPs with a final NaCl concentration of 200 and 120 mM, respectively. Centrifugation was performed to remove the non-adsorbed DNA. DNA displacement by BSA was evaluated without or with extra NaCl. Typically, HEPES buffer (100 mM, pH 7.4, 10 μL), 13 nm AuNP/DNA (10 nM, 10 μL) and BSA (10 μM, 10 μL) were mixed, and the final volume was adjusted to 100 μL followed by overnight incubation. The samples were then washed by 10 mM HEPES buffer after centrifugation for 3 times, and the collected AuNP/DNA conjugates were dissolved by 10 mM KCN in HEPES buffer (10 mM, pH 7.4) to fully release the DNA remained on the AuNPs. For the control experiment, 10 μL of HEPES buffer was added to the AuNP/DNA conjugates without BSA. The percentage of DNA desorbed by BSA was quantified by comparing the fluorescence intensity of the BSA-added and BSA-free samples. With a similar treatment, pre-adsorbed BSA displacement by DNA (A_15_ or T_15_) was also measured.

### Attachment of Thiolated DNA to AuNP/BSA Conjugates

AuNP/BSA conjugates were prepared by incubating 10 μM BSA and AuNP stock solutions for 30 min. For DNA attachment, 1 μL of 200 μM FAM-9A5-SH was added into the AuNP/BSA conjugates (10 nM AuNPs) followed by a one-time addition of 100 mM NaCl and overnight incubation (rather than increasing the concentration of NaCl gradually). For quantifying the density of attached DNA, standard curves of FAM-9A5-SH were made based on its fluorescence intensity and the UV-vis absorption spectra were recorded for determining the concentration of the AuNPs. The effect of the AuNP-to-DNA ratio (1:15; 1:30; 1:60, and 1:200) on the final DNA loading was also tested with 300 mM NaCl.

### Kinetics of Adsorption

To measure DNA adsorption kinetics, the fluorescence of 10 nM FAM-9A5-SH in 10 mM HEPES buffer (pH 7.4) was recorded in a quartz cuvette. At ~1 min, 4 μL of 10 nM AuNP/BSA conjugates or free AuNPs were added into the cuvette and the fluorescence intensity was monitored to follow the kinetics of DNA adsorption. The kinetics of BSA adsorption was monitored by recording the fluorescence of 50 nM FITC-BSA mixed with 4 μL of 10 nM AuNP solution.

### Gel Electrophoresis

The gel was prepared by dissolving 0.5% agarose in 5 mM HEPES buffer (pH 7.4). The running buffer was also 5 mM HEPES. The AuNP/DNA conjugates were obtained by traditional salt aging method. To prepare the AuNP/BSA/DNA conjugate, BSA were pre-adsorbed by mixing 10 μL BSA and 5 nM AuNP for 30 min followed by centrifugation to remove free BSA. DNA was then attached (the ratio of DNA to AuNP is 1:200) by adding 300 mM NaCl for overnight incubation. AuNP/DNA/GSH and Au/DNA/BSA conjugates were obtained by incubating 1 mM GSH or 1 μM BSA with the AuNP/DNA conjugates for 30 min. The prepared AuNP conjugates in 10 mM HEPES (pH 7.4) were mixed with an equal volume of 30% glycerol for loading into the gel. The gel was run at 90 V for 30 min and was documented by a digital camera.

### DNA Hybridization

DNA hybridization experiments were carried out by directly adding 200 nM FAM-cDNA or FAM-rDNA to a final of 1 nM (AuNP concentration) of AuNP/DNA, AuNP/protein/DNA, AuNP/DNA/BSA or AuNP/DNA/GSH (10 mM HEPES, pH 7.4, 300 mM NaCl). After overnight incubation, the samples were washed to remove the non-hybridized DNA, and KCN was then added to quantify the adsorbed FAM-labeled cDNA or rDNA. The Au/protein conjugates were obtained first by mixing 10 μM protein and 5 nM AuNPs in 10 mM HEPES for 30 min and then free protein was removed by centrifugation. Then, 1 μM DNA and 300 mM NaCl were added followed by overnight incubation. For comparison, AuNP/DNA/GSH and Au/DNA/BSA conjugates were also used, which were obtained by incubating GSH (0, 10, 100, 1,000, and 5,000 μM) or 1 μM BSA with DNA/AuNP conjugates for 30 min. The hybridization kinetics were recorded on a Cary Eclipse fluorometer.

## Results and Discussion

### Protein-Capping Increases the Colloidal Stability of AuNPs

**Figure 1:**
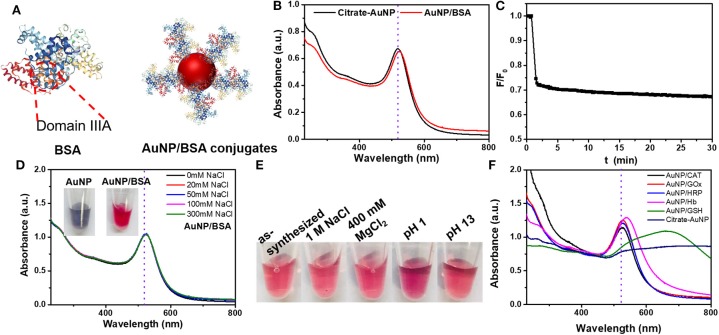
**(A)** The structures of BSA and AuNP/BSA conjugates. **(B)** The UV–vis spectra of citrate-capped AuNP and AuNP/BSA conjugates. **(C)** Adsorption kinetics of FITC-labeled BSA (50 nM) by 13 nm AuNPs (0.4 nM added at 1 min). **(D)** The UV–vis spectra of AuNP/BSA conjugates prepared by mixing 13 nm AuNPs with 10 μM BSA in up to 300 mM NaCl. Inset: photographs citrate-AuNPs with 50 mM NaCl (left), and AuNP/BSA with 300 mM NaCl. **(E)** Photographs of AuNP/BSA in different solutions. **(F)** The UV–vis spectra of AuNPs capped with various proteins conjugates in 300 mM NaCl. All samples were in HEPES buffer (10 mM, pH 7.4).

BSA is the most commonly used protein for surface blocking (An and Zhang, 2017; Chen et al., 2017; Nguyen et al., 2018), and it was also used for blocking gold surfaces (Dominguez-Medina et al., 2013; Tebbe et al., 2015; Bolaños et al., 2019). While most previous work focused on bulk gold surfaces, for AuNPs, the effect of BSA adsorption has yet to be systemically studied to gain fundamental information, such as the adsorption stability and kinetics, and the effect on colloidal stability. BSA has an approximate dimension of 9 × 9 × 4 nm^3^ with a cuboid shape, and it may use the IIIA subdomain for adsorption onto AuNPs (Figure 1A; Chaudhary et al., 2014).

After mixing BSA with the 13 nm AuNPs, a 4 nm red shift of the 520 nm plasmon peak was observed, which can be attributed to BSA changing the local dielectric constant (i.e., the localized surface plasmon resonance effect; Figure 1B). The size of the AuNPs increased by about ~9 nm from DLS, and the zeta-potential increased by 10 mV (Figures S1A,B), also confirming the adsorption of BSA.

We then employed FITC-labeled BSA to study the adsorption kinetics, and ~31% quenching was observed in the first minute upon adding the AuNPs, after which the fluorescence did not change much. Since the inner filter effect of AuNPs can also decrease the fluorescence, the sample was centrifuged and 23% of the BSA was adsorbed by measuring the fluorescence decrease in the supernatant, confirming adsorption. Therefore, BSA can be quickly adsorbed onto AuNPs (Figure 1C). From a quantitative experiment (Figure S1C), about 58 BSA molecules were adsorbed onto each 13 nm AuNP. For comparison, each AuNP can adsorb around 100 thiolated DNA (Liu and Liu, 2017a). Since BSA is much larger than DNA, such a high loading can only be explained multilayer adsorption (Dominguez-Medina et al., 2013). BSA has an isoelectric point of 4.7, and the adsorption was further promoted by lowering the pH below it, which can be attributed to the electrostatic attraction effect with negatively charged AuNPs (Figure S4).

We then examined the colloidal stability of the AuNPs. The AuNP/BSA conjugates survived up to 1 M NaCl, 400 mM MgCl_2_ and extreme pH conditions (Figures 1D,E), while the as-synthesized citrate-capped AuNPs aggregated with just 50 mM NaCl (Figure S2). BSA can have a steric stabilization effect, since the overall charge density decreased by the BSA corona (thus weaker electrostatic stabilization). The protection effect of BSA is general, and it also worked for larger AuNPs (Figure S3). Since BSA can be oxidized, we also tested BSA that was aged for more than a day. The resulting AuNP conjugates had poor colloidal stability, although the adsorption kinetics remained fast (Figure S5). It could be that upon oxidation, disulfide bonds were formed and AuNPs were then adsorbed via weaker interactions instead of the strong cysteine residues.

We further tested a few other proteins, including catalase (CAT), glucose oxidase (GOx), horse radish peroxidase (HRP), hemoglobin (Hb), and a small peptide, glutathione (GSH). They were all able to protect the colloidal stability of the AuNPs in 300 mM NaCl except for GSH (Figures 1F, S6). GSH had a weaker protection effect due to its small size (Wu et al., 2019), further attesting the importance of steric stabilization. These proteins have different isoelectric points and molecular weights (Table 2). Therefore, proteins can in general adsorb on AuNPs and render a protection effect, and protein coating is a simple and convenience for improving the colloidal stability of AuNPs even in extreme environment. Here, we in this work focused on BSA because it is cost-effective and extensively studied.

**Table 2:** The molecular weights and isoelectric points of the proteins.

**Protein**	**M.W**.	**Isoelectric point**
BSA	~66 kDa	4.7
CAT	~250 kDa	5.4
GOx	~160 kDa	4.2
HRP	~44 kDa	7.2
Hb	~64,500 Da	6.8
GSH	307 Da	5.9

### Proteins Cannot Displace DNA From AuNPs

Adsorption of DNA oligonucleotides by AuNPs has been extensively studied (Zhang et al., 2012c; Liu and Liu, 2019). DNA adsorption is relatively simple since there are only four types of nucleobases, whereas proteins are more complex with twenty types of amino acids. An interesting question is the relative adsorption strength of DNA and protein. Taking advantage of the fluorescence quenching property of AuNPs, we designed the following displacement experiments. We employed two DNA strands here: FAM-A_15_ and FAM-T_15_ to represent high and low affinity DNA on gold, respectively (Liu et al., 2018b). These two DNAs were, respectively, mixed with the AuNPs and adsorbed via salt aging. These non-thiolated DNAs were expected to adsorb lengthwise on the AuNP surface (Pei et al., 2012).

**Figure 2:**
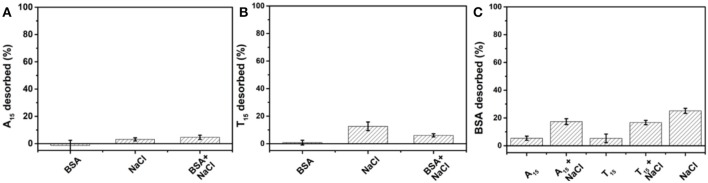
Desorbed **(A)** A_15_ and **(B)** T_15_ from their AuNP conjugates after overnight incubation with 100 mM NaCl, 1 μM BSA, or their mixture. **(C)** Desorbed BSA to each AuNP conjugate after overnight incubation with 100 mM NaCl, 1 μM A_15_, T_15_, or their combinations. All samples were in HEPES buffer (10 mM, pH 7.4).

To these AuNP/DNA conjugates, non-labeled BSA was added and incubated overnight. After washing by centrifugation, we quantified the DNA remained on the AuNPs by adding KCN. Without NaCl, adding 1 μM BSA alone desorbed almost no DNA, indicating that the DNA was highly stably adsorbed (Figure 2A). Adding BSA in the presence of 100 mM NaCl desorbed ~5% DNA, while 100 mM NaCl alone also desorbed ~2% FAM-A_15_ and ~10% FAM-T_15_ (Figure 2B). Therefore, BSA was essentially incapable of displacing the pre-adsorbed DNA, consistent with the notion of highly stable DNA adsorption by AuNPs.

On the other hand, previous work showed that non-thiolated DNA can be easily displaced by various anions and small molecules (Liu et al., 2018b; Wu et al., 2019; Zhang et al., 2019; Zong et al., 2019). The reason that BSA cannot displace DNA is attributed its large size making it difficult to penetrate through the DNA layer. Since thiolated DNA is adsorbed even stronger and can reach an even higher density, their displacement by proteins could be even more challenging. We can conclude that damages to DNA/AuNP conjugates in biological samples are unlikely from direct competition by proteins.

### DNA Cannot Displace Proteins From AuNPs

We also prepared AuNPs adsorbed with FITC-labeled BSA. For this conjugate, 100 mM NaCl alone desorbed 25% BSA (Figure 2C). This might be due to salt can help the adsorbed BSA collapse on the AuNPs, which increased the footprint of each adsorbed protein, leading to some desorption. Adding A_15_ DNA alone almost had no effect. When A_15_ and 100 mM NaCl were added together, the desorbed BSA was similar to that from 100 mM NaCl alone, also indicating that A_15_ can hardly desorb the adsorbed BSA. A similar phenomenon was observed with T_15_ DNA. Moreover, we incubated the AuNP/BSA conjugates in 100 mM NaCl overnight, and then performed the displacement experiment. Still, no displacement was observed with A_15_ or T_15_ (Figure S7).

We then added FAM-A_15_ and FAM-T_15_ to the AuNP/BSA conjugates, but no DNA adsorbed (Figure S8A), further explaining why DNA cannot displace the adsorbed BSA (Fu et al., 2014). The free cysteine, positively charged lysine residues and hydrophobic binding domains can contribute to the tight adsorption (Dominguez-Medina et al., 2013), while electrostatic interactions and van der Waals interactions are believed to be important in the initial adsorption process (Dominguez-Medina et al., 2013). The molecular weight of BSA is about 66 kDa, much higher than that of a 15-mer DNA (~4.5 k), which may also contribute strong polyvalent adsorption interactions especially for the collapsed state. Overall, both BSA and non-thiolated DNA strongly adsorbed onto AuNPs surface and they can hardly displace each other.

We previously studied the adsorption of DNA and proteins on metal oxides and graphene oxide (Liu et al., 2018a). Between these two materials, we concluded that DNA can be more tightly adsorbed on metal oxide since this adsorption relied on the phosphate backbone of DNA, and most proteins do not phosphate groups. For GO, however, DNA can be easily displaced by BSA proteins, likely due to the higher molecular weight of the protein. Gold represents a different type of surface with a very strong affinity for both proteins and DNA. In this case, the kinetic effect is critical, and molecules adsorbed first have an advantage, although the adsorbed molecules may not be thermodynamically more stable. For such kinetically controlled systems, desorption or displacement has a too high of activation energy barrier that cannot be achieved under ambient conditions.

### Adsorption Kinetics of Thiolated DNA on BSA-Capped AuNPs

**Figure 3:**
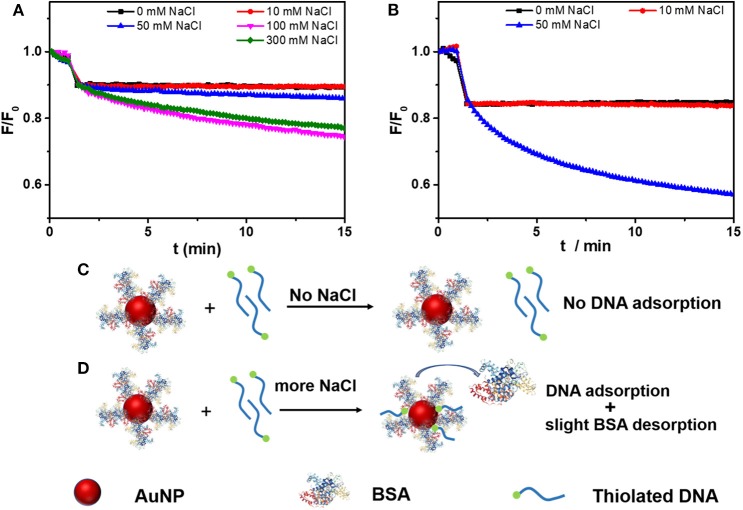
Adsorption kinetics of FAM-labeled thiolated DNA (FAM-9A5-SH) on **(A)** AuNP/BSA, and **(B)** citrate-capped AuNPs in 10 mM HEPES buffer (pH 7.4) containing various concentrations of NaCl. The AuNP/BSA conjugates were added at ~1 min. Schematic representation for thiolated DNA adsorption onto AuNP/BSA **(C)** without and **(D)** with NaCl.

After studying non-thiolated DNA, we then tested thiolated DNA. To the AuNP/BSA conjugates, a thiol and FAM dual-labeled 21-mer DNA (FAM-9A5-SH) was used for exploring the kinetics of DNA adsorption (Figure 3A). The initial quick drop was attributed to the inner filter effect of the AuNPs, after which the slow drop in fluorescence was attributed to DNA adsorption. Therefore, the thiol label enabled adsorption of the DNA with the help of NaCl, while the above non-thiolated DNA failed to adsorb on the AuNP/BSA conjugates. Non-thiolated DNA can only be attached via the DNA bases (Storhoff et al., 2002; Kimura-Suda et al., 2003), which are weaker than the thiol adsorption, leading to the inhibited non-thiolated DNA adsorption by the BSA layer.

Without NaCl, the thiolated DNA can hardly adsorb to the AuNP/BSA conjugates either (Figure 3C), and NaCl promoted the adsorption (Figure 3D). The function of NaCl can be attributed to screening the charge of DNA. Moreover, compared with the citrate-capped AuNPs (Figure 3B), the adsorption of thiolated DNA onto AuNP/BSA conjugates was slower in 50 mM NaCl (50 mM NaCl, 0.4 nM AuNPs, 10 nM FAM-9A5-SH), which indicated that the BSA corona retarted the attachment of thiolated DNA.

### Conjugation of Thiolated DNA

**Figure 4:**
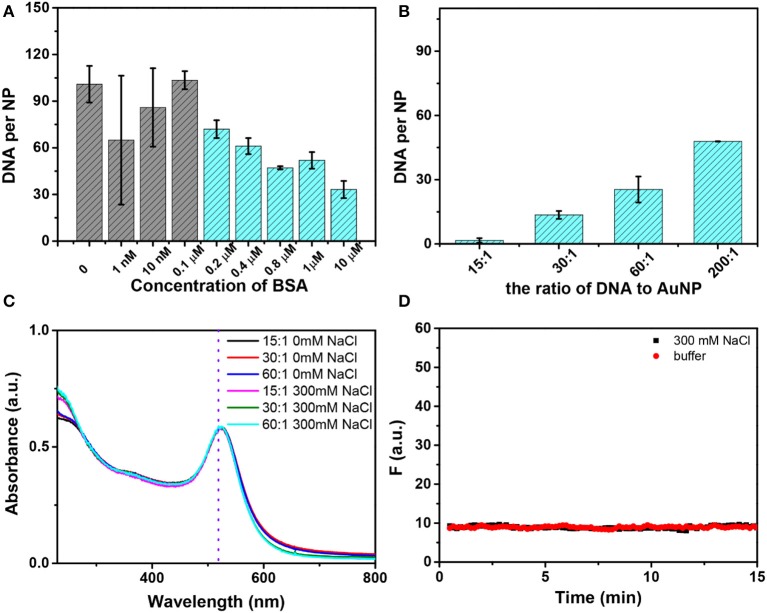
**(A)** The number of DNA strands on each AuNP as a function of the initial concentration of BSA with 100 mM NaCl. The first four samples showed purple color and the rest showed red color. **(B)** Effect of the ratio (DNA to AuNP) on DNA loading with the addition of 300 mM NaCl, and all of these samples were stable. **(C)** The UV–vis spectra of AuNP/BSA/DNA conjugates without or with the addition of NaCl. The conjugates were prepared with different ratios of AuNP/BSA to DNA. **(D)** No thiolated FAM-DNA desorbed from AuNP/BSA/DNA conjugates induced by buffer or 300 mM NaCl.

Since BSA can stabilize AuNPs and thiolated DNA can still be adsorbed, we wondered if we can use BSA to help conjugation of thiolated DNA. The conjugates with a low density of BSA showed poor colloidal stability in 1 M NaCl (Figure S9). When the concentration of BSA reached 1 μM (the ratio of AuNP to BSA being 1:200), the AuNP/BSA conjugates showed good stability as indicated by their red color (Figure S9). We prepared AuNPs mixed with various concentrations of BSA and thiolated DNA attachment (FAM-9A5-SH) was performed by a one-time addition of 100 mM NaCl. The more BSA added, the less DNA attached (Figure 4A). The final loaded DNA density can also be adjusted by the changing the initial DNA concentration (Figure 4B). Similar thiolated DNA adsorption happened on AuNP/HRP and AuNP/GOx conjugates, while no DNA adsorption occurred on AuNP/Hb or AuNP/CAT (Figure S8B), and these proteins might fully block the AuNP surface.

Based on this work, we proposed a mechanism of thiolated DNA adsorption onto AuNP/BSA conjugates. Without NaCl addition, the AuNP/BSA conjugates were so stable that free non-thiolated DNA cannot displace the BSA and no bare AuNP surface was exposed for DNA adsorption (Figure 3C). NaCl can effectively screen the charge repulsion and facilitate DNA attachment (Zhang et al., 2012b), which may be the main factor. Meanwhile, salt may change the conformation of BSA and chloride may compete with the protein, both leading to the partial desorption of BSA to make room for the thiolated DNA (Casals et al., 2010; Dominguez-Medina et al., 2013) (Figure 3D).

The colloidal stability after DNA attachment was also very high, and no obvious change was observed in 300 mM NaCl (Figure 4C). It should be pointed out that conjugates with an ultralow density of DNA (for an initial ratio of 15 DNA to 1 AuNP, only ~2 DNA strands attached) can also survive a high concentration of salt due to the BSA corona, which is beneficial for monofunctionalized AuNPs in practical applications (Hermes et al., 2012; Wang et al., 2015; Chen et al., 2016; Liu et al., 2016). Moreover, we have tested the stability of AuNP/BSA/DNA conjugates by recording the desorption kinetics of the FAM-DNA with the addition of 300 mM NaCl or buffer (Figure 4D). The data show that AuNP/BSA/DNA conjugates are highly stable.

### DNA Hybridization on AuNPs With a Protein Corona

With BSA adsorbed on the AuNPs, the DNA strands were more sparsely arranged on the surface, which may help DNA hybridization since the steric effect of neighboring DNA might be low (Liu et al., 2018b). However, BSA itself may pose a steric hindrance to prevent hybridization. To test the effect of BSA on hybridization, we used a fluorescently labeled complementary DNA (FAM-cDNA) to hybridize with the immobilized thiolated DNA (SH-9A5). As a control, a random sequenced non-complementary DNA (FAM-rDNA) was also used. The thiolated DNA was attached to the AuNPs in the presence of BSA as described above, and about eight BSA molecules were attached to each AuNP/DNA conjugate (named AuNP/DNA/BSA, Figure S1C). The density of DNA was about 40 for both BSA-free and BSA-containing AuNPs.

**Figure 5:**
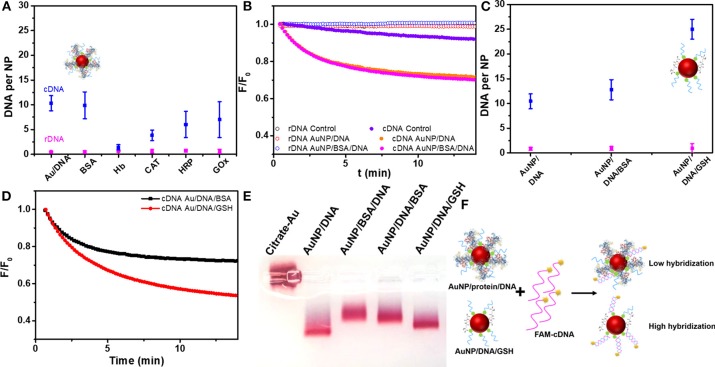
**(A)** The number of cDNA or rDNA hybridized to AuNP/DNA containing various protein corona. **(B)** The cDNA and rDNA hybridization kinetics on AuNP/DNA and AuNP/BSA/DNA. Control experiments were performed by adding citrate-AuNP for confirming the decrease of fluorescence from DNA hybridization instead from the inner-filter effect of AuNPs. **(C)** The number of cDNA or rDNA hybridized to AuNP/DNA dispersed in BSA and GSH solutions. **(D)** The cDNA and rDNA hybridization kinetics to AuNP/DNA in BSA and GSH solutions. **(E)** AuNPs conjugates analyzed by 0.5% agarose gel electrophoresis in 5 mM HEPES buffer, and the DNA was 9A5-SH. **(F)** Scheme of hybridization of AuNP/protein/DNA and AuNP/DNA/GSH conjugates.

As Figure 5A shown, the BSA layer had almost no effect on DNA hybridization. We also measured the hybridization kinetics by recording the fluorescence of cDNA (10 nM, 300 mM NaCl) after adding the AuNP/DNA conjugate (0.4 nM) (Figure 5B), and similar phenomena were observed. As a control, FAM-rDNA (a non-complementary random sequence) had no obvious change of fluorescence. For AuNPs modified with other proteins, however, lower hybridization efficiency was observed in some of them (e.g., CAT and GOx; Figure 5A). The DNA loading density determined the number of cDNA that can hybridize, which is the main factor. Other factors, such as protein size, smaller size is more favorable for DNA hybridization (*vide infra*). Therefore, the effect of protein on DNA hybridization was complex, but we have not observed any protein that significantly promoted the hybridization, suggesting the steric hindrance from the adsorbed proteins was important.

### Effect of BSA and GSH in Solution on Hybridization

The most commonly encountered case is to disperse AuNP/DNA conjugates in protein-containing samples. To mimic this process, we prepared AuNP/DNA conjugates without BSA, but BSA was added later. A smaller peptide, GSH was used for comparison since the intracellular GSH concentration is quite high. Interestingly, GSH significantly improved hybridization efficiency while BSA still showed almost no effect (Figures 5C,D). GSH may displace non-specifically adsorbed DNA bases to force an upright conformation just like the function of MCH or Br^−^ (Liu et al., 2018b). The highest hybridization capacity was obtained by incubation the DNA/AuNP conjugate with 1 mM GSH, while treatment with 5 mM GSH decreased the hybridization efficiency (Figure S10). Likely, a high concentration of GSH displaced the DNA by competing with the gold-thiol bond (Wu et al., 2019).

To further understand the effect of protein on the AuNP/DNA conjugate, we performed a gel electrophoresis experiment (Figure 5E). Citrate-capped AuNPs (lane 1) did not migrate likely due to limited charge density on the particle surface. The second lane with AuNP/DNA had the highest mobility. When BSA was first adsorbed on the AuNPs before adding the thiolated DNA (lane 3), the mobility was slower than adding the DNA first (lane 4). Therefore, adding the BSA later had less effect on the property of the AuNP/DNA conjugate. Finally, the sample with GSH added to the AuNP/DNA conjugate had a similar mobility as the untreated AuNP/DNA. Likely, attaching large BSA to the conjugate retarded the migration, while small GSH had less effect. All the samples remained red indicating stability of the conjugates, and this experiment also told us about the interaction between the protein/peptide and the AuNP/DNA conjugates. Overall, for a protein corona modification, pre-adsorbed BSA coating layer cannot help DNA hybridization, while a small peptide (e.g. GSH) as a backfilling reagent significantly enhanced hybridization (Figure 5F).

## Conclusions

In summary, we have systematically explored the effect of BSA as a stabilizer of AuNPs and obtained AuNP/BSA conjugates that can survive 1 M NaCl or 400 mM MgCl_2_ and even extremely acidic and basic environments. This stabilization effect was general to other proteins and larger AuNPs. Displacement of pre-adsorbed proteins by non-thiolated DNA was very difficult and *vice versa*, indicating that both proteins and DNA were tightly adsorbed on the AuNPs and the systems were under a strong kinetic control. DNA or proteins adsorbed first have an advantage, regardless of their thermodynamic adsorption stability.

Thiolated DNA can be controllably attached to AuNP/BSA conjugates with a one-time addition of a high concentration of salt. Thanks to the protection effect of BSA, no salt-aging steps were required. The DNA density can be controlled by changing the salt concentration, the initial BSA density, and the DNA-to-AuNP ratio during incubation. Desorption of BSA and adsorption of thiolated DNA may happen simultaneously during DNA attachment. Furthermore, the BSA layer on the AuNPs did not accelerate DNA hybridization, some other proteins even inhibited hybridization, while GSH enhanced the hybridization, suggesting that the size of protein is crucial for the function of the attached DNA. The work provided a simple, controlled method to obtained stable AuNP/BSA/DNA conjugates using a protein corona as a stabilizer, and it also revealed interesting biointerfaces on AuNPs for adsorbing DNA and proteins.

## Data Availability Statement

The datasets generated for this study are available on request to the corresponding author.

## Author Contributions

RW and HP conducted the experiments. RW, HP, L-PJ, J-JZ, and JL designed the experiments. RW, L-PJ, J-JZ, and JL wrote the manuscript.

### Conflict of Interest

The authors declare that the research was conducted in the absence of any commercial or financial relationships that could be construed as a potential conflict of interest.
