# Supplementary material for: Attaching DNA to Gold Nanoparticles With a Protein Corona
Source: Front Chem. 2020 Feb 25;8:121. doi: 10.3389/fchem.2020.00121 (PMC7052371; doi:10.3389/fchem.2020.00121)
Supplement: Supplementary file 1 [file Presentation_1.pdf]

Supplementary Material

DNA attachment to gold nanoparticles with a protein corona

Rong Wu[1,2], Huaping Peng[2,3], Jun-Jie Zhu[1], Li-Ping Jiang[1,*], and Juewen Liu[2,*]

1 State Key Laboratory of Analytical Chemistry for Life Science, School of Chemistry and Chemical Engineering, Nanjing University, Nanjing 210023, China

Email: jianglp@nju.edu.cn

2 Department of Chemistry, Waterloo Institute for Nanotechnology, University of Waterloo, Waterloo, Ontario N2L 3G1, Canada

Email: liujw@uwaterloo.ca

3 Higher Educational Key Laboratory for Nano Biomedical Technology of Fujian Province, Department of Pharmaceutical Analysis, Faculty of Pharmacy, Fujian Medical University, Fuzhou 350108, China

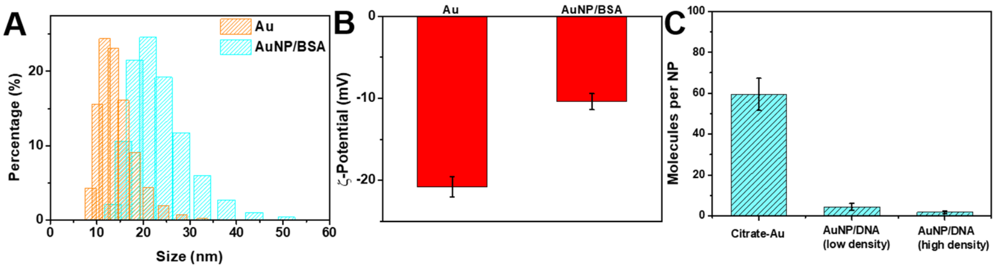

FIGURE S1 | (A) The size distribution, and (B) zeta-potential of the free 13 nm citrate-AuNPs and the AuNP/BSA conjugates. (C) The adsorbed BSA density on each AuNP. The AuNP/DNA conjugates with a low DNA loading density were obtained by mixing 1 μM DNA and 10 nM AuNP stock solution followed by the addition of 100 mM NaCl and overnight incubation. The AuNP/DNA conjugates with a high DNA loading density were prepared by the freezing method by mixing 3 μM DNA and 10 nM AuNP (Liu and Liu, 2017).

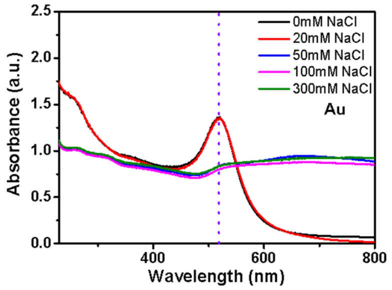

FIGURE S2 | The UV−vis spectra of the citrate-capped AuNPs after the addition of NaCl in 10mM HEPES buffer. When the concentration of NaCl reached 50 mM, the AuNPs aggregated, and a red shift can be observed in the spectra.

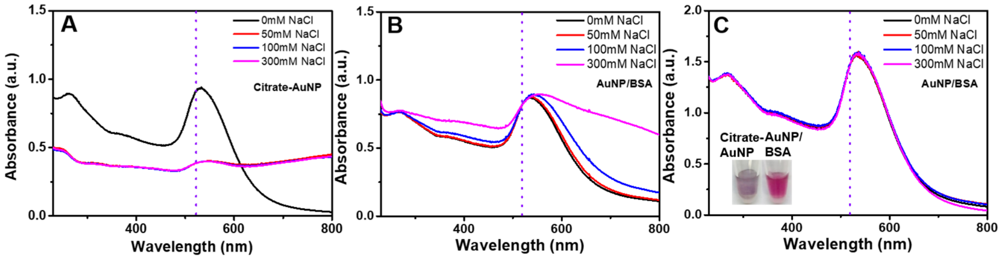

FIGURE S3 | The UV−vis spectra of 24 nm AuNPs (A) without BSA; with (B) 10 μM, and (C) 30 μM BSA after the addition of various concentrations of NaCl. To obtain stable AuNP/BSA conjugates using larger AuNPs, the concentration of BSA needs to be raised. With an optimal concentration of BSA of 30 μM, stable 24 nm AuNP/BSA conjugates were obtained and can also survive 300 mM NaCl. The inset in (C) is photographs citrate-AuNP with 50 mM NaCl (left), and AuNP/BSA conjugates with 300 mM NaCl (right).

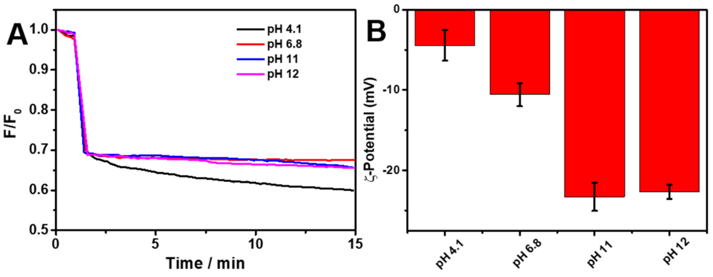

Figure S4 | (A) Adsorption kinetics of BSA at different pH. The adsorption at pH 4.1 appeared to be more favorable attributable to the partially positively charged BSA at this pH. (B) The zeta-potential of the AuNP-BSA conjugates at different pH.

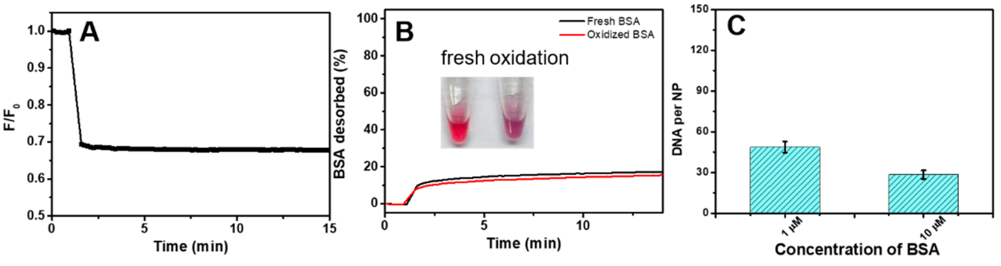

Figure S5 | (A) Adsorption kinetics of aged BSA (100 nM) by 13 nm AuNPs (0.4 nM added at 1 min). (B) Kinetics of BSA-FITC desorption from AuNP/BSA conjugates in 300 mM NaCl. (C) The number of DNA strands on each AuNP with different initial concentrations of the aged BSA.

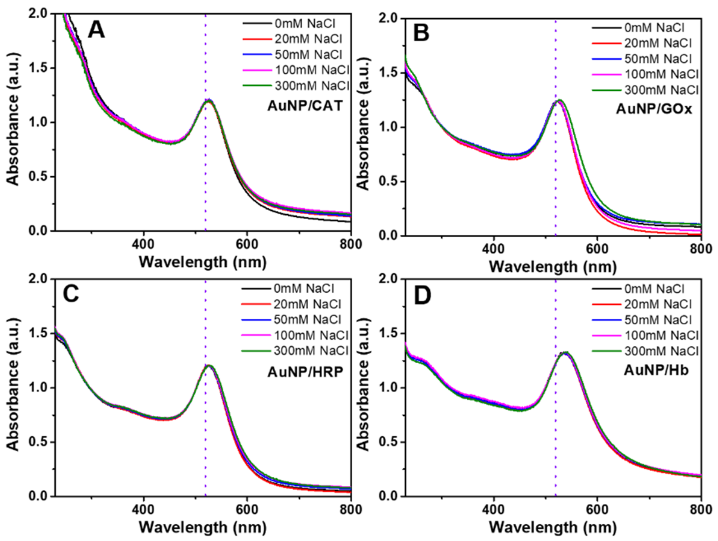

FIGURE S6 | The UV−vis spectra of (A) AuNP/CAT conjugates, (B) AuNP/GOx conjugates, (C) AuNP/HRP conjugates and (D) AuNP/Hb conjugates with the reaction concentration of 10 μM protein after the addition of various concentrations of NaCl.

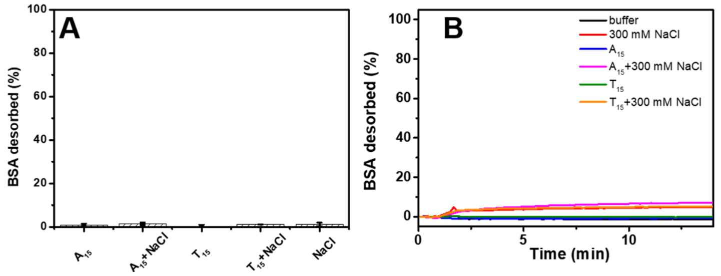

Figure S7 | (A) Desorbed BSA to each conjugate after overnight incubation with 100 mM NaCl, 1 μM A_{15}, T_{15} and their combinations. (B) BSA desorption kinetics.

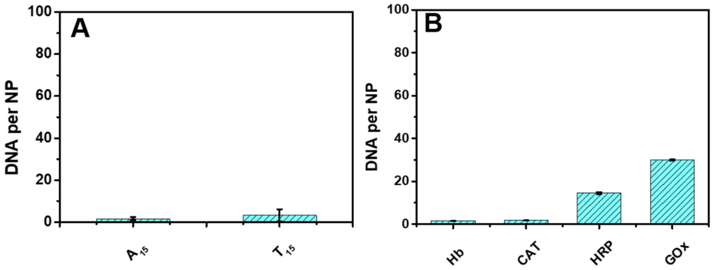

FIGURE S8 | (A) Conjugation of nonthiolated DNA onto AuNP/BSA surface. (B) The number of DNA strands on each AuNP (AuNP/protein/9A5-SH) in the presence of different proteins. The ratio of AuNP to DNA was 1:60 and the added NaCl concentration was 300 mM.

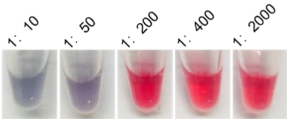

Figure S9 | Photographs showing the colloidal stability of AuNP/BSA conjugates treated by 1 M NaCl. The AuNP concentration was 5 nM, and the concentration of BSA increased from 50 nM to 10 μM.

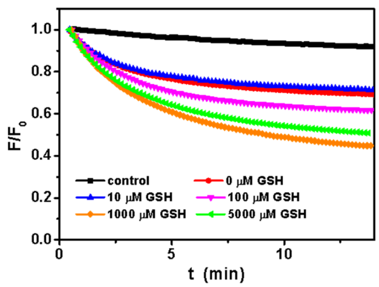

FIGURE S10 | The cDNA hybridization kinetics to the AuNP/DNA conjugates by backfilling with different concentrations of GSH.

References:

Liu, B., Liu, J., (2017) Freezing directed construction of bio/nano interfaces: reagentless conjugation, denser spherical nucleic acids, and better nanoflares. J Am Chem Soc 139, 9471–9474.doi:10.1021/jacs.7b04885
